# Association of miRNA targetome variants in LAMC1 and GNB3 genes with colorectal cancer and obesity

**DOI:** 10.1002/cam4.4713

**Published:** 2022-04-04

**Authors:** Morteza Gholami, Marziyeh Zoughi, Roobic Behboo, Reza Taslimi, Alireza Kazemeini, Milad Bastami, Shirin Hasani‐Ranjbar, Bagher Larijani, Mahsa M. Amoli

**Affiliations:** ^1^ Obesity and Eating Habits Research Center Endocrinology and Metabolism Clinical Sciences Institute, Tehran University of Medical Sciences Tehran Iran; ^2^ Metabolic Disorders Research Center Endocrinology and Metabolism Molecular‐Cellular Sciences Institute, Tehran University of Medical Sciences Tehran Iran; ^3^ Hazrate Rasoole Akram Hospital Iran University of Medical Science Tehran Iran; ^4^ Department of Gastroenterology Imam Khomeini Hospital, Tehran University of Medical Sciences Tehran Iran; ^5^ Department of General Surgery Imam Khomeini Hospital, School of Medicine, Tehran University of Medical Sciences Tehran Iran; ^6^ Department of Medical Genetics, Faculty of Medicine Tabriz University of Medical Sciences Tabriz Iran; ^7^ Endocrinology and Metabolism Research Center Endocrinology and Metabolism Clinical Sciences Institute, Tehran University of Medical Sciences Tehran Iran

**Keywords:** cancer biology, colorectal cancer, genetic variants, pathway analysis

## Abstract

**Background:**

Colorectal cancer (CRC) is one of the most common obesity‐associated cancers. Inflammation is also considered the most important factor between obesity and CRC. This study aimed to examine miRNAs binding sites variants on inflammatory genes identified using bioinformatics and systematic approach on clinical samples that were collected from CRC patients and controls.

**Methods:**

The candidate variants related to CRC inflammatory genes were obtained from genome‐wide association studies and their population‐specific haplotypes. The variants were analyzed according to their genomic position on the miRNA targetome. Targetome variants in inflammation‐related genes were selected for genetic association study by TaqMan genotyping assay.

**Results:**

The GG genotype of rs7473 decreased the risk of obesity (*p* < 0.05). Heterozygous genotype (GA) of rs1547715 decreased the risk of CRC (*p* < 0.05). In the rs7473/rs1547715 genotype and haplotype, the frequencies of AA/GA and GG/AA lessened in CRC and obesity, respectively (*p* < 0.05).

**Conclusions:**

The variants of rs7473 and rs1547715 were associated with obesity and CRC, respectively. The above‐mentioned associations could be made based on the interactions of these variants with miRNAs.

## Graphical abstract

This study aimed to examine miRNAs binding sites variants on inflammatory genes identified using bioinformatics and systematic approach on clinical samples collected from CRC patients and controls. Targetome variants in inflammation‐related genes were selected for genetic association study by TaqMan genotyping assay. The variants of rs1547715 and rs7473 are associated with CRC and obesity, respectively. These associations may be based on the interactions of these variants with miRNAs.

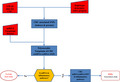

## 1 INTRODUCTION

According to the World Health Organization (WHO), the prevalence of obesity has been tripled since 1975^1^ and is estimated to be increased in pandemic dimensions over the next years.^2^, ^3^ Obesity and overweight are associated with increased cancer risk and mortality.^4^, ^5^, ^6^ Colorectal cancer (CRC) is the second most deadly cancer and third most common malignancy and the number of CRC new cases is estimated to increase in future.^7^, ^8^ In addition, CRC has been considered as the most important cancer associated with obesity and overweight.^9^, ^10^, ^11^ The above sentences highlight the need for rapid and accurate strategies to determine the connection factors of these two conditions. One of the most important commonalities between obesity and cancer is inflammation. In general, the causative agents and conditions associated with inflammation are very diverse.^12^ CRC can be associated with inflammation in different ways.^13^ The signaling pathways involved in the regeneration and repair of normal tissues are disrupted in both chronic inflammation and cancer.^14^ Therefore, inflammatory elements may cause mutations due to stimulation of cell proliferation.^12^

Several obesity‐related risk factors such as lifestyle, related syndromes, pregnancy, and sleep duration affect miRNA dysregulation^15^, ^16^, ^17^, ^18^, ^19^ which consequently regulate obesity‐associated genes or play a role in obesity‐associated cancers such as CRC.^12^, ^20^, ^21^ The genetic factors are involved in biological processes, body composition, body weight regulation, body mass index (BMI), and obesity.^22^, ^23^, ^24^, ^25^, ^26^ The variants in the miRNA binding site are potential markers of obesity and its associated cancers.^27^, ^28^, ^29^ They may alter gene expression^30^, ^31^, ^32^ or affect the risk and survival of individuals with various multifactorial diseases, such as CRC.^33^, ^34^ Most of these regions have been deeply conserved throughout evolution.^35^ Previously, we conducted a systematic review and meta‐analysis on these variants and the risk of CRC.^29^ Also, a bioinformatics study on the role of miRNAs target site interactions was conducted (miRNA:mRNA:SNP) on CRC and obesity in 2020. Genome‐wide association studies (GWAS) associated with five categories of obesity‐related traits, CRC risk, and survival were assessed. Then, bioinformatics prioritization identified separate interactions for obesity and CRC. However, no common inflammatory variant was found between obesity and CRC.^28^

Based on the high prevalence and important effect of obesity on the risk of CRC,^4^, ^9^, ^10^, ^36^, ^37^ the main role of inflammation^12^, ^38^, ^39^, ^40^ and miRNA binding sites variants on them,^27^, ^28^, ^29^ for the first time, we designed the present study to find the most common inflammatory variants and haplotypes between CRC and obesity in the miRNA binding region of Iranian CRC patients. We used literature review and bioinformatics studies to identify candidate variants in inflammatory genes and a case–control association study was designed to examine the role of selected variants and their associated haplotypes.

## 2 METHOD

### 2.1 Study design

In order to obtain shared variants between CRC and obesity, the inflammatory pathway has been selected as a common and the main mechanism involved in both conditions. For this purpose, the bioinformatics and pathway analysis approaches were applied to identify and prioritize the desired variants. Then, a case–control association study was performed on genomic DNA obtained from samples collected from CRC patients and control samples with or without obesity, respectively.

### 2.2 Study population and sampling

The study population included 215 samples in three CRC groups (CRC obese, CRC overweight, and CRC normal weight) and 215 people in three control groups (obese, overweight, and normal weight). The CRC patients were enrolled in Imam Khomeini Hospital, Tehran Clinic, and Gandhi Hospital. Control groups were also selected from those who were referred to Valiasr Laboratory of Imam Khomeini Hospital for a checkup and had no history of CRC. All study participants signed the informed consent form and 5 ml blood was taken from each subject in EDTA tubes and stored at −20°C temperature for further analysis. Written informed consent was obtained from all participants in this study. The study was approved by the Research Ethics Committee of Endocrinology and Metabolism Research Institute, Tehran University of Medical Sciences (IR.TUMS.EMRI.REC.1396.00198). The inclusion and exclusion criteria are as follows: the case and control groups were male/female over 40 years of age who signed the informed consent form and completed the questionnaire form. The WHO BMI cutoff for obesity has been applied for this study. Subjects with BMI ≥30, 25 ≤ BMI < 30, and BMI <25 were included in obesity, overweight (pre‐obesity), and normal weight groups, respectively.^1^ The people who had no history of CRC were included in the control group, while subjects with positive colonoscopy and by pathological examination after tissue biopsy for colon and/or rectal cancer were included in the CRC group. The exclusion criteria for both case and control groups include individuals less than 40 years, who decided to leave the study, pregnant women, women with infant children, people with BMI <18.5, and also people with a history of the following diseases: familial adenomatous polyposis, Lynch syndrome, Turbot syndrome, Peutz‐jeghers syndrome, MUTYH‐associated polyposis, and inflammatory bowel disease. Both medical records and questioner were used for the history of diseases. The demographic information including age, sex, BMI, family history of cancers, family history of CRC, self‐history of CRC, history of diabetes, history of smoking, high blood pressure, high blood fats, history of cardiovascular diseases, and history of thyroid disease was collected in this study.

### 2.3 Whole blood DNA extraction

DNA extraction was performed with the DNSol Kit (http://rojetechnologies.com/product/dnsol‐midi‐kit‐2/) from 2 ml of whole blood. All extraction steps were performed according to the instructions in the kit. DNA concentration was measured by NanoDrop for qualitative and quantitative analysis of DNA.

### 2.4 Identification and selection of desired variants

**FIGURE 1:**
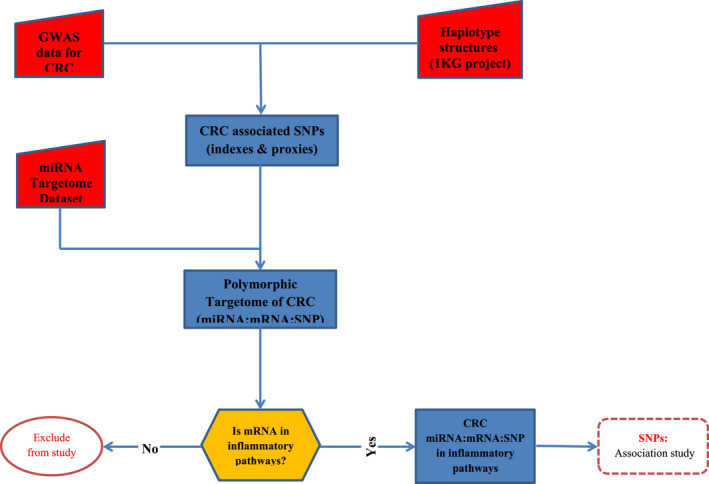
Study pipeline

An overview of the workflow is shown in Figure 1. The most important variants related to the mRNA binding sites in CRC have been identified and prioritized by integrating the different levels of available experimental data.

To identify, prioritize, and select the desired variants, the following method was used.

- Step 1: Identifying the CRC‐associated variants. For this purpose, the data of genetic association studies were obtained from the EBI‐NHGRI GWAS catalog (https://www.ebi.ac.uk/gwas/). This data includes a list of index variants identified by GWAS. Since GWAS index variants are not necessarily causative of the disease, all variants that are in the linkage disequilibrium (LD) with the index variant are considered as candidate disease variants. Therefore, to obtain all candidate variants, the GWAS information was integrated with the haplotype structures specific to phase III of the 1000Genome project, and thus the list of candidate variants, including the GWAS index and proxy variants (LD ≥0.6) were obtained.
- Step 2: Identifying the CRC miRNAs binding site variants. The binding site refers to all miRNA binding sites, along with their ±25 nucleotide regions. Therefore, the purpose of this step was to investigate which of the candidate disease‐associated variants were located in the miRNA‐mRNA binding site. For this purpose, a comprehensive set of miRNA binding sites were prepared, which includes information on binding sites confirmed by experimental studies such as CLIP‐seq. The interactions were from targetScan (version 7.1, available at http://www.targe tscan.org/vert_71/), StarBase (version 2, available at http://starb ase.sysu.edu.cn/starb ase2/index.php), and microRNA.org (available at http://www.micro rna.org/micro rna/home.do). Next, candidate variants (step one) were examined for genomic position and location at the miRNA binding site. The output of this step was a list of CRC binding site variants, which includes information on the miRNA‐mRNA interaction as well as the candidate variant at the binding site.
- Step 3: Identifying the binding site variants which were related to inflammatory pathways. From the variants identified in step 2, the variants in which the target gene was in inflammatory pathways were selected. For this purpose, the genes in inflammatory pathways were selected by using the following pathways: KEGG pathways and genecards identified pathways (Sino Biological pathway, GeneTex pathway, BioSystems pathways, Reactome pathways, PharmGKB pathways, GeneGo pathways, R&D Systems pathways, and Cell Signaling Technology pathways Qiagen pathways).
- Stage 4: The output of the third stage was entered into the experimental study, the genetic association study (case–control).

### 2.5 Assessing the association of selected variants with CRC and obesity

Genotyping of selected variants was performed by TaqMan Genotyping Assays, Applied Biosystems (Assay ID: C__3127459**_**10, Assay ID: C__26124318**_**10, Assay ID: C__1085366**_**10). The 2X master mix was purchased from Ampliqon (Taq DNA Polymerase 2× Master Mix). The reaction was performed in 10 μl volume, based on the following formula of 5 μl of 2× master mix, 0.3 μl of the primer‐probe mixture, 2 μl of DNA, and 2.7 μl of nuclease‐free water. The reaction program was 10 min at 60°C (pre‐incubation phase) and production cycles contained 10 s 95°C and 40 s 60°C for each of 40 cycles. TaqMan genotyping polymerase chain reactions (PCR) were performed in Roche LightCycler® 96 System and analyzed by LightCycler 96sw1.1 software. To confirm the accuracy of the TaqMan method, for each variant, three samples of heterozygote, wild type homozygote, and mutant homozygote had repeated TaqMan reaction. To ensure the absence of any possible contamination in the reactions, a well was assigned as the blank control without adding the DNA.

### 2.6 Statistical analysis

Statistical analysis was performed with R programming language (version 3.4.2) and IBM SPSS Statistics 22 (SPSS, Inc.). Comparisons between the two groups for continuous variables and categorical variables were performed by *t*‐test and Chi‐square (χ^2^), respectively. The deviation from the Hardy–Weinberg equilibrium was also investigated by the χ^2^ method mentioned in the previous study.^41^ The association of the selected variant with CRC and/or obesity was assessed by logistic regression. Regarding genetic models, dominant, recessive, co‐dominant, overdominant, and allelic genetic models, the odds ratio was calculated to evaluate the results of genotyping. Multivariate logistic regression analysis was used to moderate the effect of age, sex, diabetes, family history of cancers, and smoking. *p* < 0.05 was considered statistically significant.

## 3 RESULTS

### 3.1 Variants affecting the binding of miRNAs in CRC patients

The steps are shown schematically in Figure 1. First, a study was performed in the GWAS catalog of CRC patients. Based on the results, 159 variants were identified from 32 GWAS studies related to the risk of CRC, and 45 variants were related to CRC survival (with a minimum significance level of *p* < 1 × 10^−6^). In the next step, the association blocks were defined based on haplotype analysis. In this case, this information was integrated with the population‐specific haplotype structures of the 1000 Genome Project (taking into account *r* ^2^ ≥ 0.6). Finally, 5163 and 1121 candidate variants were identified for the risk of CRC and the survival rate of CRC patients, respectively. Population classification of candidate variants (proxy or index) showed that the European subgroup had the highest number of variants (4464 variants) and 2216, 130, and 53 variants were related to the Asian, American, and African populations, respectively. In the next step, the genomic position of the candidate variants was compared to the binding dataset. Identified interactions (miRNA:mRNA:SNP) associated with CRC risk or survival are shown in Data S1.

### 3.2 Interactions for genetic association study

Based on described method, the genes in CRC interactions were assessed for inflammation‐associated pathways, the results have shown that two *LAMC1* and *GNB3* variants were associated with inflammatory response and inflammation. As shown in Table 1 *LAMC1* is involved in inflammatory response pathway, *ERK* Signaling SuperPath, and *PI3K/Akt* Signaling and *GNB3* is involved in *ERK* Signaling SuperPath, *PI3K/Akt* Signaling, Signaling by *Wnt*, anti‐inflammatory response favoring Leishmania parasite infection, and *TGF‐Beta* Pathway. The *LAMC1* shows 11 target sites and 16 flanking region interactions and *GNB3* shows nine flanking region interactions that are presented in Data S2 (Table S1).

**TABLE 1:** The main inflammation‐associated pathways for included genes

Gene	Pathway	Effect on	Reference
*LAMC1* (Laminin subunit gamma 1)	Inflammatory Response Pathway	Fibrotic response	WikiPathways
	*ERK* Signaling SuperPath	*MAPK* signaling	PathCards
	*PI3K/Akt* Signaling	*PI3K* (Class IA)	KEGG
*GNB3* (G protein subunit beta 3)	*ERK* Signaling SuperPath	*MAPK* signaling	PathCards
	*PI3K/Akt* Signaling	*PI3K* (Class IB)	KEGG
	Signaling by *Wnt*	Beta‐catenin independent *Wnt* signaling	Reactome
	Antiinflammatory response favoring Leishmania parasite infection	‐	Reactome
	*TGF‐Beta* Pathway	*MAPK* Family Pathway	PathCards

### 3.3 Experimental study

The association of selected variants with obesity and CRC was considered in a genetic association study. The results are described in the following sections (demographic characteristics and association of selected variants with CRC and/or obesity).

### 3.4 Demographic characteristics

The demographic characteristics of the individuals participating in the genetic association study (*N* = 430) are given in Table 2. There was significant difference between case and control groups for age (*p* = 0.005), sex (*p* = 0.014), family history of cancers (*p* = 0.005), diabetes (*p* = 0.001), smoking (*p* = 0.001), high blood fat (*p* = 0.002), and history of thyroid disease (*p* = 0.015). There was no significant difference for BMI, family history of CRC, self‐history of CRC, high blood pressure, and history of cardiovascular diseases.

**TABLE 2:** Demographic characteristics of study participants in variants association study

Characteristics	CRC (*N* = 215)	Control (*N* = 215)	*p* value
Age (year)	58.57 ± 12.51	53.50 ± 10.15	<0.001
BMI <25	59.14 ± 16.52	55.64 ± 10.89	0.182
25 ≤ BMI < 30	60.01 ± 11.48	53.53 ± 9.70	<0.001
BMI ≥30	56.16 ± 8.80	51.27 ± 9.86	0.005
60 years (%)≤	44.19	25.12	<0.001
Sex (Male%)	53.02	41.86	0.014
BMI <25	14.88	14.42	
25 ≤ BMI < 30	24.65	23.26	
BMI ≥30	13.49	4.19	
BMI (kg/m^2^)	28.04 ± 4.77	27.59 ± 4.05	0.228
BMI <25	27.91	26.05	
25 ≤ BMI < 30	41.40	47.44	
BMI ≥30	30.70	26.51	
Family history of cancer (%)	15.81	30.23	<0.001
BMI <25	3.72	6.51	
25 ≤ BMI < 30	6.98	13.95	
BMI ≥30	5.12	9.77	
Family history to CRC (%)	5.12	3.26	0.335
BMI <25	1.86	0.47	
25 ≤ BMI < 30	1.40	0.93	
BMI ≥30	1.86	1.86	
Self‐history to CRC (%)	2.33	1.40	0.724
BMI <25	0.00	0.47	
25 ≤ BMI < 30	1.40	0.47	
BMI ≥30	0.93	0.47	
Diabetes (%)	11.16	22.79	<0.001
BMI<25	2.79	3.72	
25 ≤ BMI < 30	4.65	12.09	
BMI ≥30	3.72	6.98	
Smoking (%)	4.19	13.95	<0.001
BMI <25	0.47	6.98	
25 ≤ BMI < 30	3.26	6.05	
BMI ≥30	0.47	0.93	
High blood pressure (%)	14.88	14.88	1.000
BMI <25	4.19	0.93	
25 ≤ BMI < 30	5.58	7.44	
BMI ≥30	5.12	6.51	
High blood fat (%)	0.93	6.51	0.002
BMI <25	0.00	1.40	
25 ≤ BMI < 30	0.93	3.72	
BMI ≥30	0.00	1.40	
History of cardiovascular diseases (%)	9.77	15.81	0.061
BMI <25	3.72	5.58	
25 ≤ BMI < 30	4.19	6.98	
BMI ≥30	1.86	3.26	
History of thyroid disease (%)	4.19	10.23	0.015
BMI <25	0.93	2.33	
25 ≤ BMI < 30	1.86	5.12	
BMI ≥30	1.40	2.79	

*Note*: The age of the patient group is related to the time of diagnosis and the age of the control group is related to the time of participation in the study. Quantitative variables are specified as mean ± standard deviation and qualitative variables are specified as a percentage. For all factors, the percentage of people in each group is also reported based on BMI classification.

Abbreviations: BMI, body mass index; CRC, colorectal cancer.

### 3.5 Association of selected variants with CRC and/or obesity

To be sure of lack of contamination, no template control was used (Data S3, Figures S1 and S2) in each reaction. Also, gel electrophoresis of all three genotypes of selected variants are presented in Data S3, Figure S3). The Hardy–Weinberg equilibrium was assessed for control and different BMI groups (Table 3).

**TABLE 3:** Hardy–Weinberg equilibrium

Control groups	Overall	BMI ≥25	BMI ≥30	BMI <30	25 ≤ BMI < 30	BMI <25
rs7473	0.079	0.187	0.016	0.526	0.971	0.312
rs1547715	0.054	0.749	0.245	0.082	0.360	0.096

All subjects based on BMI	BMI <30	25 ≥ BMI < 30	BMI <25
rs7473	0.968	0.505	0.346
rs1547715	0.711	0.841	0.385

Abbreviation: BMI, body mass index.

Tables 4, 5, 6 show the genetic association of rs7473 and rs1547715 variants with CRC. According to the results, no significant relationship was found between rs7473 variant and CRC, but the result for rs1547715 in overdominant model odds ratio (OR): 0.645 (95% confidence interval [CI]; 0.42–0.99), *p* = 0.046, and also in the subgroup of overweight individuals OR: 0.497 (95% CI; 0.25–0.98), *p* = 0.044 and nonobese subjects was significant OR: 0.534 (95% CI; 0.32–0.89), *p* = 0.017 in this model. The nonobese subjects also showed significant association in comparison between GG and GA genotypes OR: 0.546 (95% CI; 0.31–0.97), *p* = 0.04. Other results, including the association of rs7473 and rs1547715 with CRC in normal weight, obese, and non‐normal weight subjects, are shown in Data S3, Tables S2–S4. All subjects were homozygote (TT) for rs5445 variant. Therefore, it was not possible to analyze the results of this variant.

**TABLE 4:** Association of rs7473 and rs1547715 with CRC

Model/variant	Genotype	CRC *N* (%)	Control *N* (%)	OR (95% CI)	*p* value
rs7473					
Dominant	AA	72 (35)	73 (35)	1.00	7.46e‐1
	GA + GG	133 (65)	136 (65)	0.930 (0.60–1.45)	
Recessive	GA + AA	174 (85)	184 (88)	1.00	2.91e‐1
	GG	31 (15)	25 (12)	1.396 (0.75–2.60)	
Overdominant	AA + GG	103 (50)	98 (47)	1.00	3.01e‐1
	AG	102 (50)	111 (53)	0.800 (0.52–1.22)	
Codominant (additive)	AA	72 (70)	73 (74)	1.00	5.16e‐1
	GG	31 (30)	25 (26)	1.259 (0.63–2.52)	
	AA	72 (41)	73 (40)	1.00	4.81e‐1
	GA	102 (59)	111 (60)	0.847 (0.53–1.35)	
	GA	102 (77)	111 (82)	1.00	2.33e‐1
	GG	31 (23)	25 (18)	1.497 (0.77–2.90)	
Allelic	A	246 (60)	257 (61)	1.00	8.61e‐1
	G	164 (40)	161 (39)	1.028 (0.76–1.40)	
rs1547715					
Dominant	GG	74 (36)	61 (31)	1.00	2.11e‐1
	GA + AA	132 (64)	138 (69)	0.750 (0.48–1.18)	
Recessive	GA + GG	172 (83)	171 (86)	1.00	2.51e‐1
	AA	34 (17)	28 (14)	1.423 (0.78–2.60)	
Overdominant	AA + GG	108 (52)	89 (45)	**1.00**	**4.6e‐2**
	AG	98 (48)	110 (55)	**0.645 (0.42–0.99)**	
Codominant (additive)	GG	74 (69)	61 (69)	1.00	6.55e‐1
	AA	34 (31)	28 (31)	1.170 (0.59–2.32)	
	GG	74 (43)	61 (36)	1.00	9.92e‐2
	GA	98 (57)	110 (64)	0.672 (0.42–1.08)	
	GA	98 (74)	110 (80)	1.00	1.14e‐1
	AA	34 (26)	28 (20)	1.681 (0.88–3.20)	
Allelic	G	246 (60)	232 (58)	1.00	7.91e‐1
	A	166 (40)	166 (42)	0.960 (0.71–1.30)	

*Note*: The OR and *p* values have been adjusted for age, sex, diabetes, family history to all cancers, and smoking.

Abbreviations: CI, confidence interval; CRC, colorectal cancer; OR, odds ratio.

The bold values are statistically significant.

**TABLE 5:** Association of rs7473 and rs1547715 with CRC in overweight subjects (25 ≤ BMI < 30)

Model/variant	Genotype	CRC *N* (%)	Control *N* (%)	OR (95% CI)	*p* value
rs7473					
Dominant	AA	33 (38)	34 (35)	1.00	9.01e‐1
	GA + GG	55 (63)	63 (65)	0.766 (0.45–1.79)	
Recessive	GA + AA	71 (81)	81 (84)	1.00	2.89e‐1
	GG	17 (19)	16 (16)	1.582 (0.68–3.70)	
Overdominant	AA + GG	50 (57)	50 (52)	1.00	2.70e‐1
	AG	38 (43)	47 (48)	0.689 (0.36–1.34)	
Codominant (additive)	AA	33 (66)	34 (68)	1.00	5.47e‐1
	GG	17 (34)	16 (32)	1.339 (0.52–3.46)	
	AA	33 (46)	34 (42)	1.00	4.98e‐1
	GA	38 (54)	47 (58)	0.777 (0.38–1.61)	
	GA	38 (69)	47 (75)	1.00	1.81e‐1
	GG	17 (31)	16 (25)	1.899 (0.74–4.86)	
Allelic	A	104 (59)	115 (59)	1.00	7.01e‐1
	G	72 (41)	79 (41)	0.701 (0.69–1.75)	
rs1547715					
Dominant	GG	35 (40)	27 (30)	1.00	2.12e‐1
	GA + AA	52 (60)	64 (70)	0.638 (0.32–1.29)	
Recessive	GA + GG	71 (82)	76 (84)	1.00	2.73e‐1
	AA	16 (18)	15 (16)	1.633 (0.68–3.93)	
Overdominant	AA + GG	51 (59)	42 (46)	**1.00**	**4.4e‐2**
	AG	36 (41)	49 (54)	**0.497 (0.25–0.98)**	
Codominant (additive)	GG	35 (69)	27 (64)	1.00	7.94e‐1
	AA	16 (31)	15 (36)	1.141 (0.43–3.06)	
	GG	35 (49)	27 (36)	1.00	7.44e‐2
	GA	36 (51)	49 (64)	0.499 (0.23–1.07)	
	GA	36 (69)	49 (77)	1.00	1.12e‐1
	AA	16 (31)	15 (23)	2.180 (0.83–5.70)	
Allelic	G	106 (61)	103 (57)	1.00	8.03e‐1
	A	68 (39)	79 (43)	0.942 (0.59–1.51)	

*Note*: The OR and *p* values have been adjusted for age, sex, diabetes, family history to all cancers, and smoking.

Abbreviations: BMI, body mass index; CI, confidence interval; CRC, colorectal cancer; OR, odds ratio.

The bold values are statistically significant.

**TABLE 6:** Association of rs7473 and rs1547715 with CRC in nonobese subjects (BMI <30)

Model/variant	Genotype	CRC *N* (%)	Control *N* (%)	OR (95% CI)	*p* value
rs7473					
Dominant	AA	52 (37)	52 (34)	1.00	7.30e‐1
	GA + GG	90 (63)	100 (66)	0.911 (0.54–1.54)	
Recessive	GA + AA	117 (82)	129 (85)	1.00	3.64e‐1
	GG	25 (18)	23 (15)	1.372 (0.69–2.72)	
Overdominant	AA + GG	77 (54)	75 (49)	1.00	3.17e‐1
	AG	65 (46)	77 (51)	0.77 (0.47–1.28)	
Codominant (additive)	AA	52 (68)	52 (69)	1.00	4.89e‐1
	GG	25 (32)	23 (31)	1.319 (0.60–2.89)	
	AA	52 (44)	52 (40)	1.00	5.23e‐1
	GA	65 (56)	77 (60)	0.836 (0.48–1.45)	
	GA	65 (72)	77 (77)	1.00	2.78e‐1
	GG	25 (28)	23 (23)	1.496 (0.72–3.10)	
Allelic	A	169 (60)	181 (60)	1.00	8.07e‐1
	G	115 (40)	123 (40)	1.046 (0.73–1. 50)	
rs1547715					
Dominant	GG	53 (38)	41 (33)	1.00	1.31e‐1
	GA + AA	88 (62)	83 (67)	0.658 (0.38–1.13)	
Recessive	GA + GG	114 (81)	122 (85)	1.00	1.91e‐1
	AA	27 (19)	22 (15)	1.583 (0.80–3.15)	
Overdominant	AA + GG	80 (57)	63 (44)	**1.00**	**1.7e‐2**
	AG	61 (43)	81 (56)	**0.534 (0.32–0.89)**	
Codominant (additive)	GG	53 (66)	41 (65)	1.00	6.33e‐1
	AA	27 (34)	22 (35)	1.214 (0.55–2.70)	
	GG	53 (46)	41 (34)	**1.00**	**4.00e‐2**
	GA	61 (54)	81 (66)	**0.546 (0.31–0.97)**	
	GA	61 (69)	81 (79)	1.00	7.4e‐2
	AA	27 (31)	22 (21)	1.943 (0.94–4.02)	
Allelic	G	167 (59)	163 (57)	1.00	7.50e‐1
	A	115 (41)	125 (43)	0.943 (0.66–1.36)	

*Note*: The OR and *p* values have been adjusted for age, sex, diabetes, family history to all cancers, and smoking.

Abbreviations: BMI, body mass index; CI, confidence interval; CRC, colorectal cancer; OR, odds ratio.

The bold values are statistically significant.

Tables 7, 8, 9 show the genetic association of rs7473 and rs1547715 variants with BMI. According to the results, no significant relationship was found between the rs1547715 variant and BMI, but the rs7473 variant showed significant association. This results were significant in recessive OR: 0.359 (95% CI; 0.16–0.80), *p* = 0.012, AA versus GG genotypes OR: 0.395 (95% CI; 0.16–0.95), *p* = 0.038, and GA versus GG genotypes OR: 0.337 (95% CI; 0.15–0.76), *p* = 0.009 models. Significant associations were also observed in comparison between obese and overweight groups. In the recessive OR: 0.309 (95% CI; 0.13–0.71), *p* = 0.006, overdominant OR: 1.76 (95% CI; 1.07–2.88), *p* = 0.025, AA versus GG genotypes OR: 0.357 (95% CI; 0.14–0.89), *p* = 0.028, and GA versus GG genotypes OR: 0.284 (95% CI; 0.12–0.67), *p* = 0.004. Also, the genetic association of the studied variants with BMI in CRC patients showed significant difference for the genotype frequency of rs7473 variant between obese and overweight subjects (AA vs. GA genotypes) OR: 0.33 (95% CI; 0.11–0.98), *p* = 0.045, and in overdominant model OR: 2.056 (95% CI; 1.02–4.16), *p* = 0.045. Other results are shown in Data S3, Tables S5–S11).

**TABLE 7:** Association of rs7473 and rs1547715 with BMI (obese vs. overweight)

Model	Genotype	Obese *N* (%)	Overweight *N* (%)	OR (95% CI)	*p* value
rs7473					
Dominant	AA	39 (34)	67 (36)	1.00	7.66e‐1
	GA + GG	75 (66)	118 (64)	1.080 (0.65–1.80)	
Recessive	GA + AA	106 (93)	152 (82)	**1.00**	**6e‐3**
	GG	8 (7)	33(18)	**0.309 (0.13–0.71)**	
Overdominant	AA + GG	47 (41)	100 (54)	**1.00**	**2.5e‐2**
	AG	67 (59)	85 (46)	**1.76 (1.07–2.88)**	
Codominant (additive)	AA	39 (83)	67 (67)	**1.00**	**2.78e‐2**
	GG	8 (17)	33 (33)	**0.357 (0.14–0.89)**	
	AA	39 (37)	67 (44)	1.00	2.27e‐1
	GA	67 (63)	85 (56)	1.391 (0.81–2.38)	
	GA	67 (89)	85 (72)	**1.00**	**3.87e‐3**
	GG	8 (11)	33 (28)	**0.284 (0.12–0.67)**	
Allelic	A	145 (64)	219 (59)	1.00	2.24e‐1
	G	83 (36)	151 (41)	0.803 (0.56–1.14)	
rs1547715					
Dominant	GG	40 (34)	62 (35)	1.00	8.85e‐1
	GA + AA	76 (66)	116 (65)	0.962 (0.58–1.61)	
Recessive	GA + GG	104 (90)	147 (83)	1.00	1.00e‐1
	AA	12 (10)	31 (17)	0.536 (0.26–1.13)	
Overdominant	AA + GG	52 (45)	93 (52)	1.00	3.07e‐1
	AG	64 (55)	85 (48)	1.291 (0.79–2.11)	
Codominant (additive)	GG	40 (77)	62 (67)	1.00	1.39e‐1
	AA	12 (23)	31 (33)	0.530 (0.23–1.23)	
	GG	40 (38)	62 (42)	1.00	7.43e‐1
	GA	64 (62)	85 (58)	1.094 (0.64–1.88)	
	GA	64 (84)	85 (73)	1.00	9.65e‐2
	AA	12 (16)	31 (27)	0.525 (0.25–1.12)	
Allelic	G	144 (62)	209 (59)	1.00	3.50e‐1
	A	88 (38)	147 (41)	0.846 (0.60–1.20)	

*Note*: The OR and *p* Values have been adjusted for age, sex, diabetes, family history to all cancers, and smoking.

Abbreviations: BMI, body mass index; CI, confidence interval; OR, odds ratio.

The bold values are statistically significant.

**TABLE 8:** Association of rs7473 and rs1547715 with BMI (obese vs. nonobese weight)

Model	Genotype	Obese *N* (%)	Non‐obese *N* (%)	OR (95% CI)	*p* value
rs7473					
Dominant	AA	39 (34)	104 (35)	1.00	8.72e‐1
	GA + GG	75 (66)	190 (65)	1.040 (0.65–1.67)	
Recessive	GA + AA	106 (93)	246 (84)	**1.00**	**1.2e‐2**
	GG	8 (7)	48 (16)	**0.359 (0.16–0.80)**	
Overdominant	AA + GG	47 (41)	152 (52)	1.00	5.4e‐2
	AG	67 (59)	142 (48)	1.565 (0.99–2.47)	
Codominant (additive)	AA	39 (83)	104 (68)	**1.00**	**3.78e‐2**
	GG	8 (17)	48 (32)	**0.395 (0.16–0.95)**	
	AA	39 (37)	104 (42)	1.00	3.46e‐1
	GA	67 (63)	142 (58)	1.265 (0.78–2.07)	
	GA	67 (89)	142 (75)	**1.00**	**9.08e‐3**
	GG	8 (11)	48 (25)	**0.337 (0.15–0.76)**	
Allelic	A	145 (64)	350 (60)	1.00	2.40e‐1
	G	83 (36)	238 (40)	0.822 (0.59–1.14)	
rs1547715					
Dominant	GG	40 (34)	94 (33)	1.00	6.58e‐1
	GA + AA	76 (66)	191 (67)	0.899 (0.56–1.44)	
Recessive	GA + GG	104 (90)	236 (83)	1.00	1.07e‐1
	AA	12 (10)	49 (17)	0.565 (0.28–1.13)	
Overdominant	AA + GG	52 (45)	143 (50)	1.00	4.62e‐1
	AG	64 (55)	142 (50)	1.184 (0.76–1.85)	
Codominant (additive)	GG	40 (77)	94 (66)	1.00	1.25e‐1
	AA	12 (23)	49 (34)	0.542 (0.25–1.19)	
	GG	40 (38)	94 (40)	1.00	9.99e‐1
	GA	64 (62)	142 (60)	1.000 (0.61–1.64)	
	GA	64 (84)	142 (74)	1.00	1.21e‐1
	AA	12 (16)	49 (26)	0.569 (0.28–1.16)	
Allelic	G	144 (62)	330 (58)	1.00	2.60e‐1
	A	88 (38)	240 (42)	0.830 (0.60–1.15)	

*Note*: The OR and *p* Values have been adjusted for age, sex, diabetes, family history to all cancers, and smoking.

Abbreviations: BMI, body mass index; CI, confidence interval; OR, odds ratio.

The bold values are statistically significant.

**TABLE 9:** Association of rs7473 and rs1547715 with BMI in CRC subjects (obese vs. overweight)

Model	Genotype	Obese *N* (%)	Overweight *N* (%)	OR (95% CI)	*p* value
rs7473					
Dominant	AA	20 (32)	33 (38)	1.00	3.91e‐1
	GA + GG	42 (68)	55 (63)	1.377 (0.66–2.86)	
Recessive	GA + AA	56 (90)	71 (81)	1.00	1.03e‐1
	GG	6 (10)	17 (19)	0.421 (0.15–1.19)	
Overdominant	AA + GG	26 (42)	50 (57)	**1.00**	**4.5e‐2**
	AG	36 (58)	38 (43)	**2.056 (1.02–4.16)**	
Codominant (additive)	AA	20 (77)	33 (66)	1.00	4.15e‐1
	GG	6 (23)	17 (34)	0.611 (0.19–2.00)	
	AA	20 (36)	33 (46)	**1.00**	**4.5e‐2**
	GA	36 (64)	38 (54)	**0.33 (0.11–0.98)**	
	GA	36 (86)	38 (69)	1.00	1.22e‐1
	GG	6 (14)	17 (31)	1.862 (0.86–4.10)	
Allelic	A	76 (61)	104 (59)	1.00	7.83e‐1
	G	48 (39)	72 (41)	0.933 (0.57–1.53)	
rs1547715					
Dominant	GG	21 (33)	35 (40)	1.00	2.73e‐1
	GA + AA	43 (67)	52 (60)	1.493 (0.73–3.06)	
Recessive	GA + GG	57 (89)	71 (82)	1.00	2.55e‐1
	AA	7 (11)	16 (18)	0.560 (0.21–1.52)	
Overdominant	AA + GG	28 (44)	51 (59)	1.00	6.1e‐2
	AG	36 (56)	36 (41)	1.951 (0.97–3.93)	
Codominant (additive)	GG	21 (75)	35 (69)	1.00	7.04e‐1
	AA	7 (25)	16 (31)	0.801 (0.26–2.51)	
	GG	21 (37)	35 (49)	1.00	1.03e‐1
	GA	36 (63)	36 (51)	1.903 (0.88–4.13)	
	GA	36 (84)	36 (69)	1.00	1.37e‐1
	AA	7 (16)	16 (31)	0.452 (0.16–1.29)	
Allelic	G	78 (61)	106 (61)	1.00	8.65e‐1
	A	50 (39)	68 (39)	1.043 (0.64–1.70)	

*Note*: The OR and *p* Values have been adjusted for age, sex, diabetes, family history to all cancers, and smoking.

Abbreviations: BMI, body mass index; CI, confidence interval; OR, odds ratio.

The bold values are statistically significant.

To investigate whether the association of the studied variants with CRC and obesity was affected by risk factors for these diseases, genotype‐risk factor interaction analysis was performed for age, sex, diabetes, family history of cancer, smoking history, high blood pressure, high blood fats, history of cardiovascular disease, history of thyroid disease were performed by different genetic models. The results demonstrated no significant interaction between the numbers of each genotype with described risk factors. The results are shown in Data S3, Tables S12 and S13.

**FIGURE 2:**
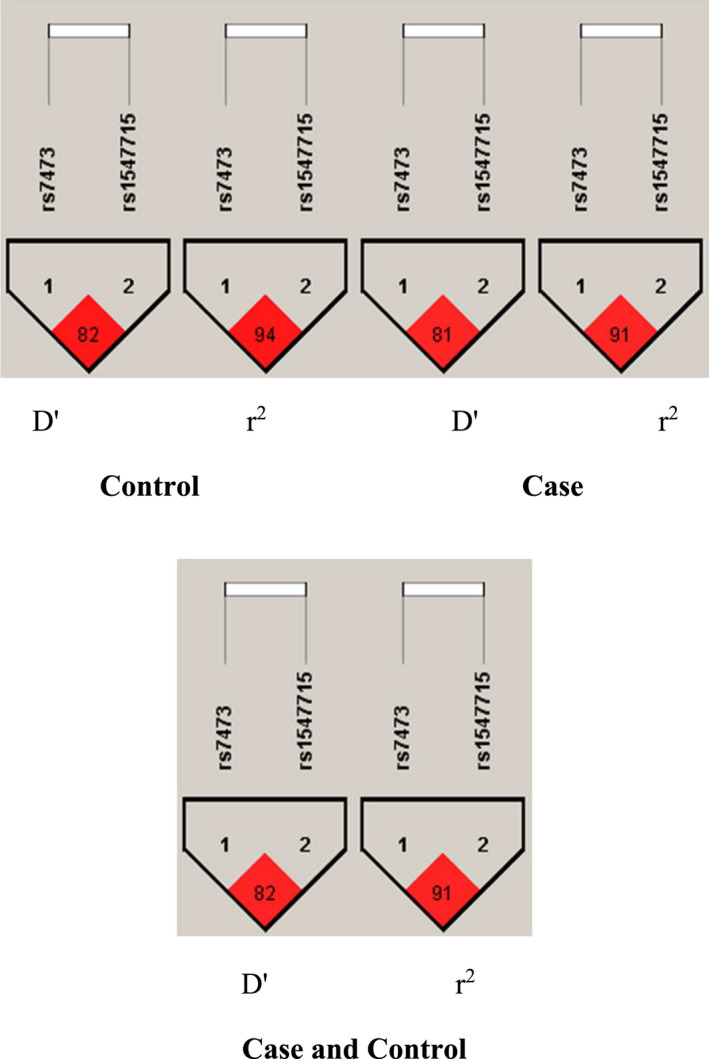
*D*′ and *r* ^2^ in overall and based on different groups

As shown in Figure 2, rs7473 and rs1547715 polymorphisms show Iranian‐specific LD blocks in CRC and control subjects with *r* ^2^ > 0.9 and *D′* > 0.8. Tables 10 and 11 show the relation between haplotypes or cumulative genotypes of rs7473/rs1547715 with CRC and obesity in Iranian subjects. Table 10 shows that AA/GA genotype frequency was significantly decreased in CRC versus control subjects (*p* < 0.05), also, GA/GA genotype frequency significantly improved in CRC obese than nonobese subjects (*p* < 0.05). As shown in Table 11, GG/AA genotype frequency was significantly reduced in obese than nonobese Iranian subjects (*p* < 0.05).

**TABLE 10:** Frequency of cumulative genotype and haplotype of rs7473/rs1547715 between CRC and control subjects

rs7473/rs154	CRC	Control	CRC normal weight	Control normal weight	CRC overweight	Control overweight	CRC obese	Control obese	CRC non‐normal weight	Control non‐normal weight	CRC non‐obese	Control non‐obese
AA/AA	0.00990	0.00000	0.01887	0.00000	0.00000	0.00000	0.01613	0.00000	0.00671	0.00000	0.00714	0.00000
AA/GA	0.00000^a^	0.03061^a^	0.00000	0.03774	0.00000	0.02273	0.00000	0.03922	0.00000	0.02878	0.00000	0.02837
AA/GG	0.34158	0.30102	0.32075	0.26415	0.39080	0.29545	0.30645	0.33333	0.35570	0.30935	0.36429	0.28369
GG/AA	0.12871	0.10204	0.13208	0.11321	0.16092	0.13636	0.08065	0.03922	0.12752	0.10072	0.15000	0.12766
GG/GA	0.01485	0.01531	0.01887	0.01887	0.02299	0.02273	0.00000	0.00000	0.01342	0.01439	0.02143	0.02128
GG/GG	0.00990	0.00000	0.00000	0.00000	0.01149	0.00000	0.01613	0.00000	0.01342	0.00000	0.00714	0.00000
GA/AA	0.01980	0.03061	0.03774	0.01887	0.02299	0.02273	0.00000	0.03922	0.01342	0.02878	0.02857	0.02128
GA/GA	0.46040	0.51020	0.45283	0.54717	0.37931^b^	0.50000	0.56452^b^ ^,^ ^c^	0.50980	0.45638	0.50360	0.40714^c^	0.51773
GA/GG	0.01485	0.01020	0.01887	0.00000	0.01149	0.00000	0.01613	0.03922	0.01342	0.01439	0.01429	0.00000
A/G	0.57861	0.57608	0.55587	0.55617	0.58596	0.55648	0.59617	0.62635	0.59016	0.58228	0.57448	0.55645
A/A	0.02040	0.03106	0.03847	0.02874	0.01174	0.02307	0.01673	0.04031	0.01387	0.02923	0.02195	0.02511
G/A	0.37564	0.37965	0.38605	0.40523	0.37332	0.40875	0.36230	0.31263	0.36868	0.37364	0.37805	0.40752
G/G	0.02535	0.01321	0.01961	0.00987	0.02898	0.01171	0.02479	0.02071	0.02729	0.01485	0.02552	0.01092
AA/G	0.34328	0.33163	0.32075	0.30189	0.39080	0.31818	0.30645	0.37255	0.35570	0.33813	0.36429	0.31206
AA/A	0.00995	0.03061	0.01887	0.03774	0.00000	0.02273	0.01613	0.03922	0.00671	0.02878	0.00714	0.02837
GG/A	0.14428	0.11735	0.15094	0.13208	0.18391	0.15909	0.08065	0.03922	0.14094	0.11511	0.17143	0.14894
GG/G	0.02488	0.01531	0.01887	0.01887	0.03448	0.02273	0.01613	0.00000	0.02685	0.01439	0.02857	0.02128
GA/A	0.48259	0.54082	0.49057	0.56604	0.40230	0.52273	0.56452	0.54902	0.46980	0.53237	0.43571	0.53901
GA/G	0.47761	0.52041	0.47170	0.54717	0.39080^d^	0.50000	0.58065^d^ ^,^ ^e^	0.54902	0.46980	0.51799	0.42143^e^	0.51773
A/AA	0.02970	0.03061	0.05660	0.01887	0.02299	0.02273	0.01613	0.03922	0.02013	0.02878	0.03571	0.02128
A/GA	0.46040	0.54082	0.45283	0.58491	0.37931^f^	0.52273	0.56452^f^ ^,^ ^g^	0.54902	0.45638	0.53237	0.40714^g^ ^,^ ^h^	0.54610^h^
A/GG	0.35644	0.31122	0.33962	0.26415	0.40230	0.29545	0.32258	0.37255	0.36913	0.32374	0.37857	0.28369
G/GG	0.02475	0.01020	0.01887	0.00000	0.02299	0.00000	0.03226	0.03922	0.02685	0.01439	0.02143	0.00000
G/GA	0.47525	0.52551	0.47170	0.56604	0.40230	0.52273	0.56452	0.50980	0.46980	0.51799	0.42857	0.53901
G/AA	0.14851	0.13265	0.169811	0.13208	0.18391	0.15909	0.08065	0.07843	0.14094	0.12950	0.17857	0.14894

Abbreviations: CI, confidence interval; CRC, colorectal cancer; OR, odds ratio.

^a^ *p* value: 0.014, OR: 1.03 (1.01–1.06), Fisher's Exact Test.

^b^ *p* value: 0.025, OR: 2.121 (1.09–4.12), Pearson Chi‐Square.

^c^ *p* value: 0.038, OR: 1.89 (1.03–3.46), Pearson Chi‐Square.

^d^ *p* value: 0.022, OR: 2.158 (1.11–4.19), Pearson Chi‐Square.

^e^ *p* value: 0.037, OR: 1.90 (1.04–3.48), Pearson Chi‐Square.

^f^ *p* value: 0.025, OR: 2.121 (1.09–4.12), Pearson Chi‐Square.

^g^ *p* value: 0.038, OR: 1.89 (1.03–3.46), Pearson Chi‐Square.

^h^ *p* value: 0.020, OR: 0.57 (0.36–0.92), Pearson Chi‐Square.

**TABLE 11:** Cumulative genotype and haplotype of rs7473/rs1547715 based on BMI

rs7473/rs1547715	Normal weight	Overweight	Obese	Normal weight	Non‐obese
AA/AA	0.00943	0.00000	0.00885	0.00347	0.00356
AA/GA	0.01887	0.01143	0.01770	0.01389	0.01423
AA/GG	0.29245	0.34286	0.31858	0.33333	0.32384
GG/AA	0.12264	0.14857^a^	0.06195^a^ ^,^ ^b^	0.11458	0.13879^b^
GG/GA	0.01887	0.02286	0.00000	0.01389	0.02135
GG/GG	0.00000	0.00571	0.00885	0.00694	0.00356
GA/AA	0.02830	0.02286	0.01770	0.02083	0.02491
GA/GA	0.50000	0.44000	0.53982	0.47917	0.46263
GA/GG	0.00943	0.00571	0.02655	0.01389	0.00712
A/G	0.55600	0.57102	0.60978	0.58642	0.56540
A/A	0.03362	0.01755	0.02738	0.02122	0.02357
G/A	0.39562	0.39102	0.33987	0.37114	0.39280
G/G	0.01475	0.02041	0.02296	0.02122	0.01823
AA/G	0.31132	0.35429	0.33628	0.34722	0.33808
AA/A	0.02830	0.01143	0.02655	0.01736	0.01779
GG/A	0.14151	0.17143^c^	0.06195^c^ ^,^ ^d^	0.12847	0.16014^d^
GG/G	0.01887	0.02857	0.00885	0.02083	0.02491
GA/A	0.52830	0.46286	0.55752	0.50000	0.48754
GA/G	0.50943	0.44571^e^	0.56637^e^	0.49306	0.46975
A/AA	0.03774	0.02286	0.02655	0.02431	0.02847
A/GA	0.51887	0.45143	0.55752	0.49306	0.47687
A/GG	0.30189	0.34857	0.34513	0.34722	0.33096
G/GG	0.00943	0.01143	0.03540	0.02083	0.01068
G/GA	0.51887	0.46286	0.53982	0.49306	0.48399
G/AA	0.15094	0.17143^f^	0.07965^f^ ^,^ ^g^	0.13542	0.16370^g^

Abbreviations: BMI, body mass index; CI, confidence interval; OR, odds ratio.

^a^ *p* value: 0.024, OR: 0.38 (0.16–0.90), Pearson Chi‐Square.

^b^ *p* value: 0.032, OR: 0.41 (0.18–0.95), Pearson Chi‐Square.

^c^ *p* value: 0.007, OR: 0.32 (0.14–0.75), Pearson Chi‐Square.

^d^ *p* value: 0.009, OR: 0.35 (0.15–0.79), Pearson Chi‐Square.

^e^ *p* value: 0.046, OR: 1.62 (1.01–2.62), Pearson Chi‐Square.

^f^ *p* value: 0.026, OR: 0.42 (0.19–0.92), Pearson Chi‐Square.

^g^ *p* value: 0.029, OR: 0.45 (0.21–0.94), Pearson Chi‐Square.

## 4 DISCUSSION

Previously a bioinformatics study was conducted on the role of miRNAs targetome variants and identified candidate variants that may separately be associated with CRC or obesity.^28^ The current study adds knowledge to the previously published study by focusing on inflammation as a common factor between CRC and obesity, for the first time, the new candidate rs5445, rs1547715, and rs7473 variants on inflammatory interactions (genes and miRNAs) were identified and validated in this study, which highlights the role of common inflammatory variants between these diseases.

rs5445 is located on the *GNB3* gene. The expression of *GNB3* changed in TCGA CRC tumor tissues compared to control tissues.^42^ The previous studies showed that *GNB3* is in network interactions for CRC.^43^ Also, a few studies investigated some variants of this gene in different types of cancers.^44^, ^45^ While the associations of our selected variants were not assessed in patients with CRC or other types of cancers, however, literature review and analysis of GWAS results in our study revealed their importance. Previous studies investigated the association of this gene and its polymorphisms with obesity.^46^, ^47^ The wild‐type allele of the rs5445 variant was observed in this study. In the 1000 Genome project, the frequency of the mutant allele of this variant was 0.16 and in African American and Asian populations, the frequency of this allele was between 0.14 and 0.32, indicating the allelic abundance of this variant is highly influenced by race.^48^ However, the mutant allele ratio was 0.005 in the European population, which is similar to our results.

The results for the rs7473 variant indicated that the genetic association of this variant with obesity was significant. The results revealed that GG genotype significantly reduces the risk of obesity. In comparison between different subgroups in CRC patients, the risk of CRC was significantly higher in obese people with heterozygous genotype (GA) than lower weight people. These variants are located at the 3′UTR of the *LAMC1* gene. *LAMC1* expression is studied in the tissue of some types of cancers including, hepatocellular or cervical carcinomas.^49^, ^50^, ^51^ It has been reported that the association of this gene with CRC and its interaction with miR‐506 has an inhibitory effect on the malignancy of CRC cells.^52^ The result of rs1547715 revealed a lower risk for CRC in people with heterozygous genotype (GA) than other genotypes. Fascinating results were obtained in comparing the frequency of cumulative genotypes (rs7473/rs1547715) with CRC and obesity. In general, these variants showed strong LD blocks in both case and control groups in Iranian subjects. Also, the genotype frequencies of AA/GA in the cancer group showed a significant decrease, and in relation to BMI, the genotype frequency of GG/AA in the obese group was decreased in the Iranian population. In the previous studies, the relationship between rs6695837, rs1062044, and rs10911251 polymorphisms of *LAMC1* and CRC was investigated.^53^, ^54^, ^55^ However, to the best of our knowledge, no studies so far have been performed on the relationship between our selected variant with CRC, obesity, or other diseases. This shows the novelty of our results and eliminates the ability to compare our results with the previous studies. However, our results might be associated with the effect of this variant on the role of *LAMC1* gene in CRC. These variants, located in miRNA binding sites, can exert their effect by changing the contact and regulatory effects of specific miRNA to *LAMC1* and lead to a change in *LAMC1* expression.

In conclusion, rs1547715 may be effective on CRC, and GG genotype of rs7473 variant is effective in reducing the risk of obesity in the Iranian population. These variants are in LD in the Iranian population. In the cumulative rs7473/rs1547715 genotypes, the frequency of AA/GA in the CRC group and the frequency of GG/AA in the obese group were significantly decreased. At last, such results provide a better understanding of the common genetic association between these two diseases. For the first time we investigated and validated the role of inflammatory rs1547715 and rs7473 variants on the risk of CRC /and or obesity. These novel results highlight the importance of inflammatory variants, their haplotypes as common genetic risk markers between cancer and obesity.

### 4.1 Strengths and limitations

This study for the first time focused on inflammation as shared feature to find CRC and obesity‐associated common variants. The results were also adjusted based on different risk factors. However, there were some limitations as described below. We only studied the role of inflammatory variants on CRC and obesity, however, the mechanism of association of these variants and their role on genes and miRNAs binding should be investigated in future studies. This study only assessed the association of selected variants in small population, therefore further studies on other populations and larger samples are recommended.

## CONFLICT OF INTEREST

The authors declare no conflict of interest.

## AUTHOR CONTRIBUTIONS

All authors contributed to the study conception and design. Roobic Behboo, Reza Taslimi, Alireza Kazemeini, and Shirin Hasani‐Ranjbar provided clinical data about patients. Morteza Gholami, Marziyeh Zoughi, and Mahsa M. Amoli carried out the experiment. The data collection and analysis were performed by Morteza Gholami, Milad Bastami, and Bagher Larijani. The first draft of the manuscript was written by Morteza Gholami and Marziyeh Zoughi with support from Mahsa M.Amoli and all authors commented on previous versions of the manuscript and discussed the results. Mahsa M. Amoli supervised the project and approved the final version. All authors read and approved the final manuscript.

## ETHICS STATEMENT

The study was approved by the Research Ethics Committee of Endocrinology and Metabolism Research Institute, Tehran University of Medical Sciences (IR.TUMS.EMRI.REC.1396.00198).

## Supporting information


Data S1

Data S2

Data S3

## Data Availability

The data that supports the findings of this study are available in the supplementary material of this article
