# Supplementary material for: Association of miRNA targetome variants in LAMC1 and GNB3 genes with colorectal cancer and obesity
Source: Cancer Med. 2022 Apr 4;11(21):3923–38. doi: 10.1002/cam4.4713 (PMC9636511; doi:10.1002/cam4.4713)
Supplement: Supplementary file 2 — Data S2 [file CAM4-11-3923-s003.docx]

The interactions associated to inflammatory selected genes Table S1.

| **LAMC1** | |
| --- | --- |
| **Target site(11)** | **Flanking region(16)** |
| hsa-miR-150:LAMC1:rs1547715  hsa-miR-150-5p:LAMC1:rs1547715  hsa-miR-423-3p:LAMC1:rs1547715  hsa-miR-216a:LAMC1:rs944970  hsa-miR-216a-5p:LAMC1:rs944970  hsa-miR-33-5p:LAMC1:rs20557  hsa-miR-33a:LAMC1:rs20557  hsa-miR-33b:LAMC1:rs20557  hsa-miR-371-5p:LAMC1:rs2027076  hsa-miR-376c:LAMC1:rs2027078  hsa-miR-590-3p:LAMC1:rs6424890 | hsa-miR-33a-5p:LAMC1:rs7473  hsa-miR-506-3p:LAMC1:rs7473  hsa-miR-124-3p:LAMC1:rs7473  hsa-miR-15a-5p:LAMC1:rs3359  hsa-miR-16-5p:LAMC1:rs3359  hsa-miR-24-3p:LAMC1:rs944971  hsa-miR-424-5p:LAMC1:rs3359  hsa-miR-497-5p:LAMC1:rs3359  hsa-miR-140-5p:LAMC1:rs6424890  hsa-miR-203a:LAMC1:rs1051473  hsa-miR-195-5p:LAMC1:rs3359  hsa-miR-320a:LAMC1:rs6424890  hsa-miR-320b:LAMC1:rs6424890  hsa-miR-320c:LAMC1:rs6424890  hsa-miR-422a:LAMC1:rs3359  hsa-miR-15b:LAMC1:rs3359 |
| **GNB3(9)** | |
| **Flanking region(9)** | |
| hsa-miR-183:GNB3:rs5445  hsa-miR-183:GNB3:rs5445  hsa-miR-124:GNB3:rs5445  hsa-miR-150:GNB3:rs5445  hsa-miR-506:GNB3:rs5445  hsa-miR-24:GNB3:rs11288917  hsa-miR-24a:GNB3:rs11288917  hsa-miR-24b:GNB3:rs11288917  hsa-miR-24-3p:GNB3:rs11288917 | |
