# Supplementary material for: Association of miRNA targetome variants in LAMC1 and GNB3 genes with colorectal cancer and obesity
Source: Cancer Med. 2022 Apr 4;11(21):3923–38. doi: 10.1002/cam4.4713 (PMC9636511; doi:10.1002/cam4.4713)
Supplement: Supplementary file 3 — Data S3 [file CAM4-11-3923-s002.docx]

**rs5445-Homo VIC**

**
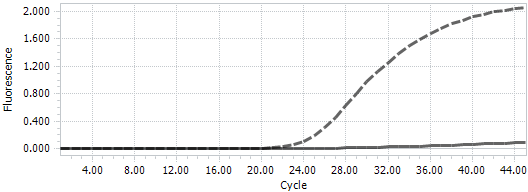

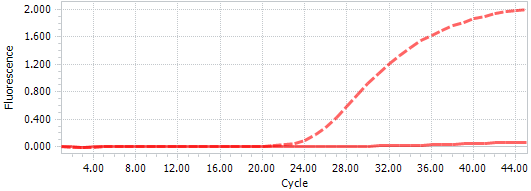
**

**rs7473-Hetero**

**
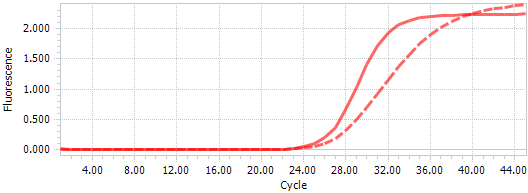

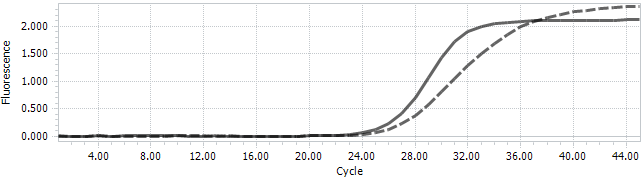
**

**rs1547715-Homo FAM**

**
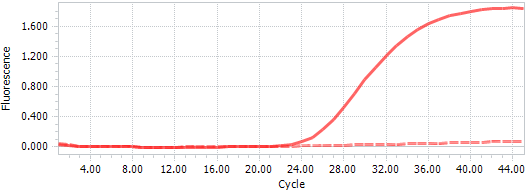

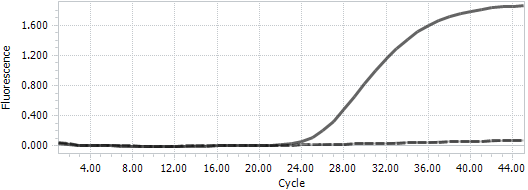
**

**Figure S1. This figure shows tree repeated samples. Continuous lines are FAM probe and dotted lines are VIC.**

**rs1547715-Heterozygote +NTC**


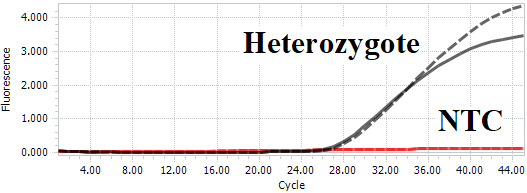


**rs7473-Heterozygote+NT**


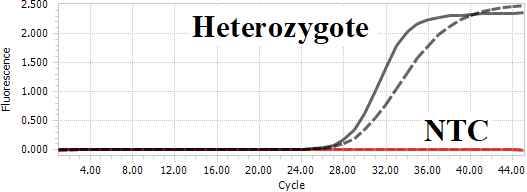


**rs5445-Homozygote+NTC**


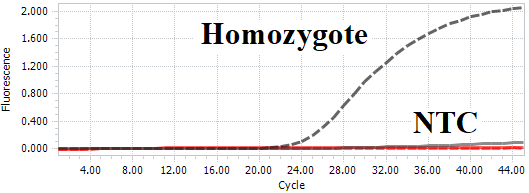


**Figure S2. The no template control (NTC) vs. genotypes**

**
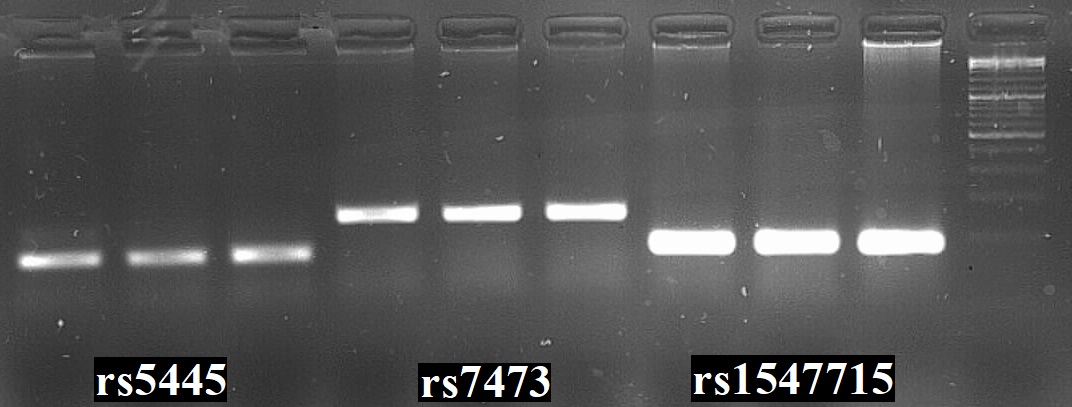
**

**Figure S3. Gel electrophoresis for three genotypes of each variant**

**Table S2. Association of rs7473 and rs1547715 with CRC in normal weight subjects (BMI˂25)**

| ***P* Value** | **OR**  **(95%CI)** | **Control**  **N.(%)** | **CRC**  **N(%)** | **Genotype** | **Model/variant** |
| --- | --- | --- | --- | --- | --- |
|  |  |  |  |  | **rs7473** |
| 7.86e-1 | 1.00 | 18(33) | 19(35) | AA | Dominant |
|  | 0.883(0.36-2.17) | 37(67) | 35(65) | GA+GG |  |
| 7.12e-1 | 1.00 | 48(87) | 46(85) | GA+AA | Recessive |
|  | 1.266(0.36-4.42) | 7(13) | 8(15) | GG |  |
| 6.04e-1 | 1.00 | 25(45) | 27(50) | AA+GG | Overdominant |
|  | 0.796(0.34-1.88) | 30(55) | 27(50) | AG |  |
| 8.39e-1 | 1.00 | 18(72) | 19(70) | AA | Codominant (adittive) |
|  | 1.168(0.26-5.21) | 7(28) | 8(30) | GG |  |
| 7.34e-1 | 1.00 | 18(38) | 19(41) | AA |  |
|  | 0.849(0.33-2.18) | 30(63) | 27(59) | GA |  |
| 7.85e-1 | 1.00 | 30(81) | 27(77) | GA |  |
|  | 1.201(0.32-4.48) | 7(19) | 8(23) | GG |  |
| 9.99e-1 | 1.00 | 66(60) | 65(61) | A | Allelic |
|  | 1.000(0.54-1.83) | 44(40) | 41(39) | G |  |
|  |  |  |  |  | **rs1547715** |
| 4.14e-1 | 1.00 | 14(26) | 18(33) | GG | Dominant |
|  | 0.675(0.26-1.73) | 39(74) | 36(67) | GA+AA |  |
| 3.45e-1 | 1.00 | 46(87) | 43(80) | GA+GG | Recessive |
|  | 1.79(0.54-5.98) | 7(13) | 11(20) | AA |  |
| 1.45e-1 | 1.00 | 21(40) | 29(54) | AA+GG | Overdominant |
|  | 0.524(0.22-1.25) | 32(60) | 25(46) | AG |  |
| 6.73e-1 | 1.00 | 14(67) | 18(62) | GG | Codominant (adittive) |
|  | 1.380(0.31-6.17) | 7(33) | 11(38) | AA |  |
| 2.84e-1 | 1.00 | 14(30) | 18(42) | GG |  |
|  | 0.585(0.22-1.56) | 32(70) | 25(58) | GA |  |
| 3.16 e-1 | 1.00 | 32(82) | 25(69) | GA |  |
|  | 1.908(0.54-6.75) | 7(18) | 11(31) | AA |  |
| 9.89e-1 | 1.00 | 60(57) | 61(56) | G | Allelic |
|  | 0.972(0.54-1.80) | 46(43) | 47(44) | A |  |

The odds ratios (OR) and *P* Values have been adjusted for age, sex, diabetes, family history to all cancers and smoking.

**Table S3. Association of rs7473 and rs1547715 with CRC in obese subjects (BMI≥30)**

| ***P* Value** | **OR**  **(95%CI)** | **Control**  **N.(%)** | **CRC**  **N(%)** | **Genotype** | **Model/variant** | |
| --- | --- | --- | --- | --- | --- | --- |
|  |  |  |  |  | **rs7473** | |
| 6.48e-1 | 1.00 | 19(37) | 20(32) | AA | Dominant | |
|  | 0.814(0.34-1.97) | 33(63) | 42(68) | GA+GG |  |  |
| 3.10e-1 | 1.00 | 50(96) | 56(90) | GA+AA | Recessive | |
|  | 2.581(0.41-16.09) | 2(4) | 6(10) | GG |  |  |
| 3.35e-1 | 1.00 | 21(40) | 26(42) | AA+GG | Overdominant | |
|  | 0.653(0.28-1.55) | 31(60) | 36(58) | AG |  |  |
| 4.11e-1 | 1.00 | 19(90) | 20(77) | AA | Codominant (adittive) | |
|  | 2.317(0. 31-17.16) | 2(10) | 6(23) | GG |  |  |
| 5.43e-1 | 1.00 | 19(38) | 20(36) | AA |  |  |
|  | 0.757(0.31-1.85) | 31(62) | 36(64) | GA |  |  |
| 3.22e-1 | 1.00 | 31(94) | 36(86) | GA |  |  |
|  | 2.679(0.38-18.79) | 2(6) | 6(14) | GG |  |  |
| 9.52e-1 | 1.00 | 69(66) | 76(61) | A | Allelic | |
|  | 1.019(0.54-1.91) | 35(34) | 48(39) | G |  |  |
|  |  |  |  |  | **rs1547715** |  |
| 9.17e-1 | 1.00 | 19(37) | 21(33) | GG | Dominant |  |
|  | 0.845(0.39-2.17) | 33(63) | 43(67) | GA+AA |  |  |
| 6.66e-1 | 1.00 | 47(90) | 57(89) | GA+GG | Recessive |  |
|  | 1.356(0.34-5.41) | 5(10) | 7(11) | AA |  |  |
| 6.48e-1 | 1.00 | 24(46) | 28(44) | AA+GG | Overdominant |  |
|  | 0.822(0.35-1.91) | 28(54) | 36(56) | AG |  |  |
| 6.43e-1 | 1.00 | 19(79) | 21(75) | GG | Codominant (adittive) |  |
|  | 1.432(0.31-6.55) | 5(21) | 7(25) | AA |  |  |
| 8.09e-1 | 1.00 | 19(40) | 21(37) | GG |  |  |
|  | 0.897(0.37-2.16) | 28(60) | 36(63) | GA |  |  |
| 6.86e-1 | 1.00 | 28(85) | 36(84) | GA |  |  |
|  | 1.385(0.29-6.70) | 5(15) | 7(16) | AA |  |  |
| 9.57e-1 | 1.00 | 66(63) | 78(61) | G | Allelic |  |
|  | 1.017(0.55-1.88) | 38(37) | 50(39) | A |  |  |

The odds ratios (OR) and *P* Values have been adjusted for age, sex, diabetes, family history to all cancers and smoking.

**Table S4. Association of rs7473 and rs1547715 with CRC in non-normal weight subjects (BMI≥25)**

| ***P* Value** | **OR**  **(95%CI)** | **Control**  **N.(%)** | **CRC**  **N(%)** | **Genotype** | **Model/variant** |
| --- | --- | --- | --- | --- | --- |
|  |  |  |  |  | **rs7473** |
| 5.79e-1 | 1.00 | 53(36) | 53(35) | AA | Dominant |
|  | 0.860(0.51-1.46) | 96(64) | 97(65) | GA+GG |  |
| 3.37e-1 | 1.00 | 131(88) | 127(85) | GA+AA | Recessive |
|  | 1.43(0.69-2.97) | 18(12) | 23(15) | GG |  |
| 2.29e-1 | 1.00 | 71(48) | 76(51) | AA+GG | Overdominant |
|  | 0.730(0.44-1.22) | 78(52) | 74(49) | AG |  |
| 6.04e-1 | 1.00 | 53(75) | 53(70) | AA | Codominant (adittive) |
|  | 1.236(0.55-2.76) | 18(25) | 23(30) | GG |  |
| 4.10e-1 | 1.00 | 53(40) | 53(42) | AA |  |
|  | 0.792(0.46-1.38) | 78(60) | 74(58) | GA |  |
| 2.14e-1 | 1.00 | 78(81) | 74(76) | GA |  |
|  | 1.652(0.75-3.65) | 18(19) | 23(24) | GG |  |
| 9.19e-1 | 1.00 | 184(62) | 180(60) | A | Allelic |
|  | 1.019(0.71-1.47) | 114(38) | 120(40) | G |  |
|  |  |  |  |  | **rs1547715** |
| 2.68e-1 | 1.00 | 46(32) | 56(37) | GG | Dominant |
|  | 0.739(0.43-1.26) | 97(68) | 95(63) | GA+AA |  |
| 3.96e-1 | 1.00 | 123(86) | 128(85) | GA+GG | Recessive |
|  | 1.367(0.67-2.81) | 20(14) | 23(15) | AA |  |
| 9.60e-2 | 1.00 | 66(46) | 79(52) | AA+GG | Overdominant |
|  | 0.644(0.38-1.08) | 77(54) | 72(48) | AG |  |
| 8.28e-1 | 1.00 | 46(70) | 56(71) | GG | Codominant (adittive) |
|  | 1.093(0.50-2.43) | 20(30) | 23(29) | AA |  |
| 1.52e-1 | 1.00 | 46(37) | 56(44) | GG |  |
|  | 0.663(0.38-1.16) | 77(63) | 72(56) | GA |  |
| 1.93e-1 | 1.00 | 77(79) | 72(76) | GA |  |
|  | 1.689(0.77-3.72) | 20(21) | 23(24) | AA |  |
| 7.43e-1 | 1.00 | 169(59) | 184(61) | G | Allelic |
|  | 0.940(0.65-1.36) | 117(41) | 118(39) | A |  |

The odds ratios (OR) and *P* Values have been adjusted for age, sex, diabetes, family history to all cancers and smoking.

**Table S5. Association of rs7473 and rs1547715 with BMI (overweight vs. normal weight)**

| *P* Value | OR  (95%CI) | Normal weight  N.(%) | Owerweight  N.(%) | Genotype | Model |
| --- | --- | --- | --- | --- | --- |
|  |  |  |  |  | **rs7473** |
| 5.77e-1 | 1.00 | 37(34) | 67(36) | AA | Dominant |
|  | 0.867(0.52-1.43) | 72(66) | 118(64) | GA+GG |  |
| 3.84-1 | 1.00 | 94(86) | 152(82) | GA+AA | Recessive |
|  | 1.346(0.69-2.63) | 15(14) | 33(18) | GG |  |
| 2.39e-1 | 1.00 | 52(48) | 100(54) | AA+GG | Overdominant |
|  | 0.749(0.46-1.21) | 57(52) | 85(46) | AG |  |
| 6.03e-1 | 1.00 | 37(71) | 67(67) | AA | Codominant (adittive) |
|  | 1.221(0.58-2.60) | 15(29) | 33(33) | GG |  |
| 3.88e-1 | 1.00 | 37(39) | 67(44) | AA |  |
|  | 0.791(0.47-1.35) | 57(61) | 85(56) | GA |  |
| 3.06e-1 | 1.00 | 57(79) | 85(72) | GA |  |
|  | 1.446(0.71-2.93) | 15(21) | 33(28) | GG |  |
| 4.92e-1 | 1.00 | 131(66) | 219(59) | A | Allelic |
|  | 0.886(0.63-1.25) | 67(34) | 151(41) | G |  |
|  |  |  |  |  | **rs1547715** |
| 3.03e-1 | 1.00 | 32(30) | 62(35) | GG | Dominant |
|  | 0.759(0.45-1.28) | 75(70) | 116(65) | GA+AA |  |
| 9.90e-1 | 1.00 | 89(83) | 147(83) | GA+GG | Recessive |
|  | 1.00(0.53-1.92) | 18(17) | 31(17) | AA |  |
| 3.27e-1 | 1.00 | 50(47) | 93(52) | AA+GG | Overdominant |
|  | 0.784(0.48-1.28) | 57(53) | 85(48) | AG |  |
| 6.58e-1 | 1.00 | 32(64) | 62(67) | GG | Codominant (adittive) |
|  | 0.845(0.40-1.78) | 18(36) | 31(33) | AA |  |
| 2.90e-1 | 1.00 | 32(36) | 62(42) | GG |  |
|  | 0.742(0.43-1.29) | 57(64) | 85(58) | GA |  |
| 6.81e-1 | 1.00 | 57(76) | 85(73) | GA |  |
|  | 1.154(0.58-2.28) | 18(24) | 31(27) | AA |  |
| 7.50e-1 | 1.00 | 121(57) | 209(59) | G | Allelic |
|  | 0.943(0.66-1.36) | 93(43) | 147(41) | A |  |

The odds ratios (OR) and *P* Values have been adjusted for age, sex, diabetes, family history to all cancers and smoking.

**Table S6. Association of rs7473 and rs1547715 with BMI (obese vs. normal weight)**

| *P* Value | OR  (95%CI) | Normal weight  N.(%) | Obese  N.(%) | Genotype | Model |
| --- | --- | --- | --- | --- | --- |
|  |  |  |  |  | **rs7473** |
| 8.32e-1 | 1.00 | 37(34) | 39(34) | AA | Dominant |
|  | 1.066(0.59-1.93) | 72(66) | 75(66) | GA+GG |  |
| 1.55e-1 | 1.00 | 94(86) | 106(93) | GA+AA | Recessive |
|  | 0.500(0.19-1.30) | 15(14) | 8(7) | GG |  |
| 2.78e-1 | 1.00 | 52(48) | 47(41) | AA+GG | Overdominant |
|  | 1.370(0.78-2.42) | 57(52) | 67(59) | AG |  |
| 3.41e-1 | 1.00 | 37(71) | 39(83) | AA | Codominant (adittive) |
|  | 0.584(0.19-1.77) | 15(29) | 8(17) | GG |  |
| 5.38e-1 | 1.00 | 37(39) | 39(37) | AA |  |
|  | 1.212(0.66-2.24) | 57(61) | 67(63) | GA |  |
| 9.95e-2 | 1.00 | 57(79) | 67(89) | GA |  |
|  | 0.442(0.17-1.17) | 15(21) | 8(11) | GG |  |
| 6.25e-1 | 1.00 | 131(60) | 145(64) | A | Allelic |
|  | 0.904(0.60-1.36) | 87(40) | 83(36) | G |  |
|  |  |  |  |  | **rs1547715** |
| 6.36e-1 | 1.00 | 32(30) | 40(34) | GG | Dominant |
|  | 0.866(0.48-1.57) | 75(70) | 76(66) | GA+AA |  |
| 2.63e-1 | 1.00 | 89(83) | 104(90) | GA+GG | Recessive |
|  | 0.624(0.27-1.43) | 18(17) | 12(10) | AA |  |
| 7.47e-1 | 1.00 | 50(47) | 52(45) | AA+GG | Overdominant |
|  | 1.096(0.63-1.92) | 57(53) | 64(55) | AG |  |
| 3.70e-1 | 1.00 | 32(64) | 40(77) | GG | Codominant (adittive) |
|  | 0.641(0.24-1.70) | 18(36) | 12(23) | AA |  |
| 9.40e-1 | 1.00 | 32(36) | 40(38) | GG |  |
|  | 0.845(0.51-1.75) | 57(64) | 64(62) | GA |  |
| 2.70e-1 | 1.00 | 57(76) | 64(84) | GA |  |
|  | 0.619(0.26-1.45) | 18(24) | 12(16) | AA |  |
| 3.86e-1 | 1.00 | 121(57) | 144(62) | G | allelic |
|  | 0.838(0.56-1.25) | 93(43) | 88(38) | A |  |

The odds ratios (OR) and *P* Values have been adjusted for age, sex, diabetes, family history to all cancers and smoking.

**Table S7. Association of rs7473 and rs1547715 with BMI (non-normal vs. normal weight)**

| *P* Value | OR  (95%CI) | Normal  N.(%) | Non-normal  N.(%) | Genotype | Model |
| --- | --- | --- | --- | --- | --- |
|  |  |  |  |  | **rs7473** |
| 7.11e-1 | 1.00 | 37(34) | 106(35) | AA | Dominant |
|  | 0.915(0.57-1.46) | 72(66) | 193(65) | GA+GG |  |
| 9.90e-1 | 1.00 | 94(86) | 258(86) | GA+AA | Recessive |
|  | 0.996(0.52-1.90) | 15(14) | 41(14) | GG |  |
| 7.30e-1 | 1.00 | 94(62) | 147(49) | AA+GG | Overdominant |
|  | 0.924(0.59-1.45) | 57(38) | 152(51) | AG |  |
| 9.37e-1 | 1.00 | 37(71) | 106(72) | AA | Codominant (adittive) |
|  | 0.971(0.47-2.00) | 15(29) | 41(28) | GG |  |
| 7.14e-1 | 1.00 | 37(39) | 106(41) | AA |  |
|  | 0.912(0.56-1.49) | 57(61) | 152(59) | GA |  |
| 9.76e-1 | 1.00 | 57(79) | 152(79) | GA |  |
|  | 1.011(0.51-1.99) | 15(21) | 41(21) | GG |  |
| 7.93e-1 | 1.00 | 131(60) | 364(61) | A | allelic |
|  | 0.958(0.70-1.32) | 87(40) | 234(39) | G |  |
|  |  |  |  |  | **rs1547715** |
| 2.95e-1 | 1.00 | 32(30) | 102(35) | GG | Dominant |
|  | 0.771(0.48-1.25) | 75(70) | 192(65) | GA+AA |  |
| 5.83e-1 | 1.00 | 89(83) | 251(85) | GA+GG | Recessive |
|  | 0.843(0.46-1.55) | 18(17) | 43(15) | AA |  |
| 5.50e-1 | 1.00 | 50(47) | 145(49) | AA+GG | Overdominant |
|  | 0.871(0.56-1.37) | 57(53) | 149(51) | AG |  |
| 3.58e-1 | 1.00 | 32(64) | 102(70) | GG | Codominant (adittive) |
|  | 0.720(0.36-1.45) | 18(36) | 43(30) | AA |  |
| 3.49e-1 | 1.00 | 32(36) | 102(41) | GG |  |
|  | 0.784(0.47-1.31) | 57(64) | 149(59) | GA |  |
| 8.45e-1 | 1.00 | 57(76) | 149(78) | GA |  |
|  | 0.938(0.49-1.79) | 18(24) | 43(22) | AA |  |
| 3.20e-1 | 1.00 | 121(57) | 353(60) | G | allelic |
|  | 0.850(0.62-1.17) | 93(43) | 235(40) | A |  |

The odds ratios (OR) and *P* Values have been adjusted for age, sex, diabetes, family history to all cancers and smoking.

**Table S8. Association of rs7473 and rs1547715 with BMI in CRC subjects (overweight vs. normal weight)**

| *P* Value | OR  (95%CI) | Normal  N.(%) | Overweight  N.(%) | Genotype | | Model |
| --- | --- | --- | --- | --- | --- | --- |
|  |  |  |  |  | | **rs7473** |
| 9.12e-1 | 1.00 | 19(35) | 33(38) | AA | | Dominant |
|  | 1.00(0.97-1.03) | 35(65) | 55(63) | GA+GG | |  |
| 4.75e-1 | 1.00 | 46(85) | 71(81) | GA+AA | | Recessive |
|  | 1.404(0.55-3.56) | 8(15) | 17(19) | GG | |  |
| 7.92e-1 | 1.00 | 27(50) | 50(57) | AA+GG | | Overdominant |
|  | 0.792(0.40-1.59) | 27(50) | 38(43) | AG | |  |
| 6.09e-1 | 1.00 | 19(70) | 33(66) | AA | | Codominant (adittive) |
|  | 1.312(0.46-3.71) | 8(30) | 17(34) | GG | |  |
| 4.16e-1 | 1.00 | 19(41) | 33(46) | AA | |  |
|  | 1.509(0.56-4.07) | 27(59) | 38(54) | GA | |  |
| 6.94e-1 | 1.00 | 27(77) | 38(69) | GA | |  |
|  | 0.856(0.39-1.86) | 8(23) | 17(31) | GG | |  |
| 7.51e-1 | 1.00 | 65(61) | 104(59) | A | | Allelic |
|  | 1.084(0.66-1.78) | 41(39) | 72(41) | G | |  |
|  |  |  |  |  | **rs1547715** | |
| 4.93e-1 | 1.00 | 18(33) | 35(40) | GG | Dominant | |
|  | 0.774(0.37-1.61) | 36(67) | 52(60) | GA+AA |  |  |
| 6.28e-1 | 1.00 | 43(80) | 71(82) | GA+GG | Recessive | |
|  | 0.804(0.33-1.95) | 11(20) | 16(18) | AA |  |  |
| 7.77e-1 | 1.00 | 29(54) | 51(59) | AA+GG | Overdominant | |
|  | 0.904(0.45-1.82) | 25(46) | 36(41) | AG |  |  |
| 5.00e-1 | 1.00 | 18(62) | 35(69) | GG | Codominant (adittive) | |
|  | 0.708(0.26-1.93) | 11(38) | 16(31) | AA |  |  |
| 5.67e-1 | 1.00 | 18(42) | 35(49) | GG |  |  |
|  | 0.794(0.36-1.75) | 25(58) | 36(51) | GA |  |  |
| 8,61e-1 | 1.00 | 25(69) | 36(69) | GA |  |  |
|  | 0.918(0.36-2.38) | 11(31) | 16(31) | AA |  |  |
| 4.57e-1 | 1.00 | 61(56) | 106(61) | G | Allelic | |
|  | 0.827(0.50-1.36) | 47(44) | 68(39) | A |  |  |

The odds ratios (OR) and *P* Values have been adjusted for age, sex, diabetes, family history to all cancers and smoking.

**Table S9. Association of rs7473 and rs1547715 with BMI in CRC subjects (obese vs. normal weight)**

| *P* Value | OR  (95%CI) | Normal weight  N.(%) | Obese  N.(%) | Genotype | Model |
| --- | --- | --- | --- | --- | --- |
|  |  |  |  |  | **rs7473** |
| 4.72e-1 | 1.00 | 19(35) | 20(32) | AA | Dominant |
|  | 1.34(0.60-3.01) | 35(65) | 42(68) | GA+GG |  |
| 6.29e-1 | 1.00 | 46(85) | 56(90) | GA+AA | Recessive |
|  | 0.750(0.23-2.40) | 8(15) | 6(10) | GG |  |
| 3.24e-1 | 1.00 | 27(50) | 26(42) | AA+GG | Overdominant |
|  | 1.463(0.69-3.12) | 27(50) | 36(58) | AG |  |
| 6.30e-1 | 1.00 | 19(70) | 20(77) | AA | Codominant (adittive) |
|  | 1.455(0.32-6.68) | 8(30) | 6(23) | GG |  |
| 4.00e-1 | 1.00 | 19(41) | 20(36) | AA |  |
|  | 0.598(0.18-1.98) | 27(59) | 36(64) | GA |  |
| 4.25e-1 | 1.00 | 27(77) | 36(86) | GA |  |
|  | 1.404(0.61-3.23) | 8(23) | 6(14) | GG |  |
| 7.95e-1 | 1.00 | 65(61) | 76(61) | A | Allelic |
|  | 1.075(0.62-1.86) | 41(39) | 48(39) | G |  |
|  |  |  |  |  | **rs1547715** |
| 6.45e-1 | 1.00 | 18(33) | 21(33) | GG | Dominant |
|  | 1.208(0.54-2.70) | 36(67) | 43(67) | GA+AA |  |
| 2.43e-1 | 1.00 | 43(80) | 57(89) | GA+GG | Recessive |
|  | 0.536(0.19-1.53) | 11(20) | 7(11) | AA |  |
| 2.05e-1 | 1.00 | 29(54) | 28(44) | AA+GG | Overdominant |
|  | 1.623(0.77-3.43) | 25(46) | 36(56) | AG |  |
| 8.07e-1 | 1.00 | 18(62) | 21(75) | GG | Codominant (adittive) |
|  | 0.850(0.23-3.14) | 11(38) | 7(25) | AA |  |
| 4.87e-1 | 1.00 | 18(42) | 21(37) | GG |  |
|  | 1.348(0.58-3.13) | 25(58) | 36(63) | GA |  |
| 1.45e-1 | 1.00 | 25(69) | 36(84) | GA |  |
|  | 0.441(0.15-1.33) | 11(31) | 7(16) | AA |  |
| 7.62e-1 | 1.00 | 61(56) | 78(61) | G | Allelic |
|  | 0.921(0.54-1.57) | 47(44) | 50(39) | A |  |

The odds ratios (OR) and *P* Values have been adjusted for age, sex, diabetes, family history to all cancers and smoking.

**Table S10. Association of rs7473 and rs1547715 with BMI in CRC subjects (non-normal vs. normal weight)**

| *P* Value | OR  (95%CI) | Normal  N.(%) | Non-normal  N.(%) | Genotype | Model |
| --- | --- | --- | --- | --- | --- |
|  |  |  |  |  | **rs7473** |
| 8.24e-1 | 1.00 | 19(35) | 53(35) | AA | Dominant |
|  | 1.078(0.56-2.09) | 35(65) | 97(65) | GA+GG |  |
| 8.37e-1 | 1.00 | 46(85) | 127(85) | GA+AA | Recessive |
|  | 1.097(0.45-2.66) | 8(15) | 23(15) | GG |  |
| 9.52e-1 | 1.00 | 27(50) | 76(51) | AA+GG | Overdominant |
|  | 1.020(0.54-1.92) | 27(50) | 74(49) | AG |  |
| 7.00e-1 | 1.00 | 19(70) | 53(70) | AA | Codominant (adittive) |
|  | 1.218(0.45-3.33) | 8(30) | 23(30) | GG |  |
| 9.22e-1 | 1.00 | 19(41) | 53(42) | AA |  |
|  | 1.048(0.41-2.65) | 27(59) | 74(58) | GA |  |
| 6.94e-1 | 1.00 | 27(77) | 74(76) | GA |  |
|  | 0.856(0.39-1.86) | 8(23) | 23(24) | GG |  |
| 7.96e-1 | 1.00 | 65(61) | 180(60) | A | Allelic |
|  | 1.062(0.67-1.67) | 41(39) | 120(40) | G |  |
|  |  |  |  |  | **rs1547715** |
| 7.71e-1 | 1.00 | 18(33) | 56(37) | GG | Dominant |
|  | 0.906(0.47-1.76) | 36(67) | 95(63) | GA+AA |  |
| 3.55e-1 | 1.00 | 43(80) | 128(85) | GA+GG | Recessive |
|  | 0.681(0.30-1.54) | 11(20) | 23(15) | AA |  |
| 6.83e-1 | 1.00 | 29(54) | 79(52) | AA+GG | Overdominant |
|  | 1.140(0.61-2.14) | 25(46) | 72(48) | AG |  |
| 4.70e-1 | 1.00 | 18(62) | 56(71) | GG | Codominant (adittive) |
|  | 0.709(0.28-1.80) | 11(38) | 23(29) | AA |  |
| 9.38e-1 | 1.00 | 18(42) | 56(44) | GG |  |
|  | 0.972(0.48-1.98) | 25(58) | 72(56) | GA |  |
| 3.74e-1 | 1.00 | 25(69) | 72(76) | GA |  |
|  | 0.674(0.28-1.61) | 11(31) | 23(24) | AA |  |
| 4.89e-1 | 1.00 | 61(56) | 184(61) | G | Allelic |
|  | 0.853(0.54-1.34) | 47(44) | 118(39) | A |  |

The odds ratios (OR) and *P* Values have been adjusted for age, sex, diabetes, family history to all cancers and smoking.

**Table S11. Association of rs7473 and rs1547715 with BMI in CRC subjects (obese vs. non-obese weight)**

| *P* Value | OR  (95%CI) | Non-obese  N.(%) | Obese  N.(%) | Genotype | Model |
| --- | --- | --- | --- | --- | --- |
|  |  |  |  |  | **rs7473** |
| 5.19e-1 | 1.00 | 52(37) | 20(32) | AA | Dominant |
|  | 1.240(0.65-2.38) | 90(63) | 42(68) | GA+GG |  |
| 1.88e-1 | 1.00 | 117(82) | 56(90) | GA+AA | Recessive |
|  | 0.523(0.20-1.37) | 25(18) | 6(10) | GG |  |
| 1.19e-1 | 1.00 | 77(54) | 26(42) | AA+GG | Overdominant |
|  | 1.639(0.88-3.05) | 65(46) | 36(58) | AG |  |
| 5.13e-1 | 1.00 | 52(68) | 20(77) | AA | Codominant (adittive) |
|  | 0.690(0.23-2.10) | 25(32) | 6(23) | GG |  |
| 1.00e-1 | 1.00 | 52(44) | 20(36) | AA |  |
|  | 0.434(0.16-1.18) | 65(56) | 36(64) | GA |  |
| 9.18e-1 | 1.00 | 65(72) | 36(86) | GA |  |
|  | 1.037(0.52-2.08) | 25(28) | 6(14) | GG |  |
| 8.10e-1 | 1.00 | 169(60) | 76(61) | A | Allelic |
|  | 0.947(0.61-1.48) | 115(40) | 48(39) | G |  |
|  |  |  |  |  | **rs1547715** |
| 4.72e-1 | 1.00 | 53(38) | 21(33) | GG | Dominant |
|  | 1.27(0.67-2.41) | 88(62) | 43(67) | GA+AA |  |
| 2.00e-1 | 1.00 | 114(81) | 57(89) | GA+GG | Recessive |
|  | 0.551(0.22-1.37) | 27(19) | 7(11) | AA |  |
| 1.01e-1 | 1.00 | 80(57) | 28(44) | AA+GG | Overdominant |
|  | 1.674(0.91-3.10) | 61(43) | 36(56) | AG |  |
| 5.61e-1 | 1.00 | 53(66) | 21(75) | GG | Codominant (adittive) |
|  | 0.733(0.26-2.09) | 27(34) | 7(25) | AA |  |
| 2.39e-1 | 1.00 | 53(46) | 21(37) | GG |  |
|  | 1.501(0.76-2.95) | 61(54) | 36(63) | GA |  |
| 1.12e-1 | 1.00 | 61(69) | 36(84) | GA |  |
|  | 0.466(0.18-1.20) | 27(31) | 7(16) | AA |  |
| 8.51e-1 | 1.00 | 167(59) | 78(61) | G | Allelic |
|  | 0.959(0.62-1.49) | 115(41) | 50(39) | A |  |

The odds ratios (OR) and *P* Values have been adjusted for age, sex, diabetes, family history to all cancers and smoking.

**Table S12. Results of genotype-risk factor interaction analyses for rs7473 in dominant and recessive models**

| Genotype (%) | | | P interaction recessive | P interaction dominant | Group | Risk factor |
| --- | --- | --- | --- | --- | --- | --- |
| AA | AG | GG |  |  |  |  |
| 93(64) | 132(62) | 34(61) | 0.759 | 0.626 | 60˂ | Age |
| 52(36) | 81(38) | 22(39) |  |  | 60≤ |  |
| 76(52) | 110(52) | 29(52) | 0.981 | 0.886 | Female | Sex |
| 69(48) | 103(48) | 27(48) |  |  | Male |  |
| 121(83) | 176(83) | 47(84) | 0.959 | 0.857 | No | Diabetes |
| 24(17) | 37(17) | 9(16) |  |  | Yes |  |
| 105(72) | 161(76) | 38(67) | 0.213 | 0.731 | No | Family history of cancer |
| 40(28) | 51(24) | 19(33) |  |  | Yes |  |
| 129(89) | 197(92) | 51(86) | 0.264 | 0.465 | No | Smoking history |
| 16(11) | 16(8) | 8(14) |  |  | Yes |  |
| 127(88) | 181(85) | 47(84) | 0.675 | 0.432 | No | High blood pressure |
| 18(12) | 32(15) | 9(16) |  |  | Yes |  |
| 139(96) | 207(97) | 52(93) | 0.171 | 0.834 | No | High blood fats |
| 6(4) | 6(3) | 4(7) |  |  | Yes |  |
| 124(86) | 183(86) | 49(88) | 0.832 | 0.839 | No | History of cardiovascular disease |
| 21(14) | 30(14) | 7(13) |  |  | Yes |  |
| 134(92) | 200(94) | 51(91) | 0.544 | 0.734 | No | History of thyroid disease |
| 11(8) | 13(6) | 5(9) |  |  | Yes |  |

**Table S13. Results of genotype-risk factor interaction analyses for rs1547715 in dominant and recessive models**

| Genotype (%) | | | | | P interaction recessive | P interaction dominant | | Group | Risk factor |
| --- | --- | --- | --- | --- | --- | --- | --- | --- | --- |
| GG | AG | | AA | |  |  |  |  |  |
| 89(66) | 127(61) | | 36(58) | | 0.463 | 0.277 | | 60˂ | Age |
| 46(34) | 81(39) | | 26(42) | |  |  |  | 60≤ |  |
| 71(53) | 112(54) | | 30(48) | | 0.471 | 1.000 | | Female | Sex |
| 64(47) | 96(46) | | 32(52) | |  |  |  | Male |  |
| 114(84) | 174(84) | | 49(79) | | 0.339 | 0.638 | | No | Diabetes |
| 21(16) | 34(16) | | 13(21) | |  |  |  | Yes |  |
| 98(73) | 155(75) | | 41(66) | | 0.215 | 1.000 | | No | Family history of cancer |
| 37(27) | 53(25) | | 21(34) | |  |  |  | Yes |  |
| 121(90) | 195(94) | | 55(89) | | 0.372 | 0.311 | | No | Smoking history |
| 14(10) | 13(6) | | 7(11) | |  |  |  | Yes |  |
| 117(87) | 179(86) | | 48(77) | | 0.072 | 0.492 | | No | High blood pressure |
| 18(13) | 29(14) | | 14(23) | |  |  |  | Yes |  |
| 129(96) | 203(98) | | 58(94) | | 0.213 | 0.577 | | No | High blood fats |
| 6(4) | 5(2) | | 4(6) | |  |  |  | Yes |  |
| 120(88) | 182(88) | | 50(81) | | 0.112 | 0.575 | | No | History of cardiovascular disease |
| 16(12) | 25(12) | | 12(19) | |  |  |  | Yes |  |
| 126(93) | 192(92) | | 58(94) | | 0.814 | 0.785 | | No | History of thyroid disease |
| 9(7) | 16(8) | | 4(6) | |  |  |  | Yes |  |
|  | |  | |  | | |  |  |  |
